# 4-Phenyl-1,2,4-tri­aza­spiro­[4.4]non-1-ene-3-thione

**DOI:** 10.1107/S1600536814005418

**Published:** 2014-03-15

**Authors:** Joel T. Mague, Shaaban K. Mohamed, Mehmet Akkurt, Alaa A. Hassan, Mustafa R. Albayati

**Affiliations:** a Department of Chemistry, Tulane University, New Orleans, LA 70118, USA; b Chemistry and Environmental Division, Manchester Metropolitan University, Manchester M1 5GD, England; c Chemistry Department, Faculty of Science, Minia University, 61519 El-Minia, Egypt; d Department of Physics, Faculty of Sciences, Erciyes University, 38039 Kayseri, Turkey; e Kirkuk University, College of Science, Department of Chemistry, Kirkuk, Iraq

## Abstract

In the title compound, C_12_H_13_N_3_S, the 4,5-di­hydro-3*H*-1,2,4-triazole system is nearly planar [maximum deviation = 0.014 (2) Å], while the cyclo­pentane ring adopts a half-chair conformation. The dihedral angle between the mean plane of the 4,5-di­hydro-3*H*-1,2,4-triazole-3-thione ring and the phenyl ring is 85.49 (14)°, with the S atom 0.046 (1) Å out of the former plane. The crystal structure is stabilized only by van der Waals inter­actions. The investigated crystal was found to be a non-merohedral two-component twin by a 180° rotation about c*, with a refined value of the minor twin fraction of 0.12203 (18).

## Related literature   

For wide-spectrum medicinal applications of spiro compounds incorporating heterocyclic substructures, see: Sar *et al.* (2006 ▶); Park *et al.* (2007 ▶); Nakao *et al.* (2008 ▶); Obniska & Kamiński (2006 ▶); Kamiński *et al.* (2008 ▶); Obniska *et al.* (2006 ▶); Chin *et al.* (2008 ▶); Wang *et al.* (2007 ▶); Pawar *et al.* (2009 ▶); Thadhaney *et al.* (2010 ▶); (Chande *et al.*, 2005 ▶); Shimakawa *et al.* (2003 ▶); Sarma *et al.* (2010 ▶). For industrial uses of heterocyclic spiro compounds, see: Rongbao *et al.* (2009 ▶); Hu *et al.* (2006 ▶); Méhes *et al.* (2012 ▶); Billah *et al.* (2008 ▶). For the crystal structure of a similar compound, see: Akkurt *et al.* (2013 ▶). For ring-puckering parameters, see: Cremer & Pople (1975 ▶). For the indexing program for twinned crystals, see: Sheldrick (2008*a* ▶).
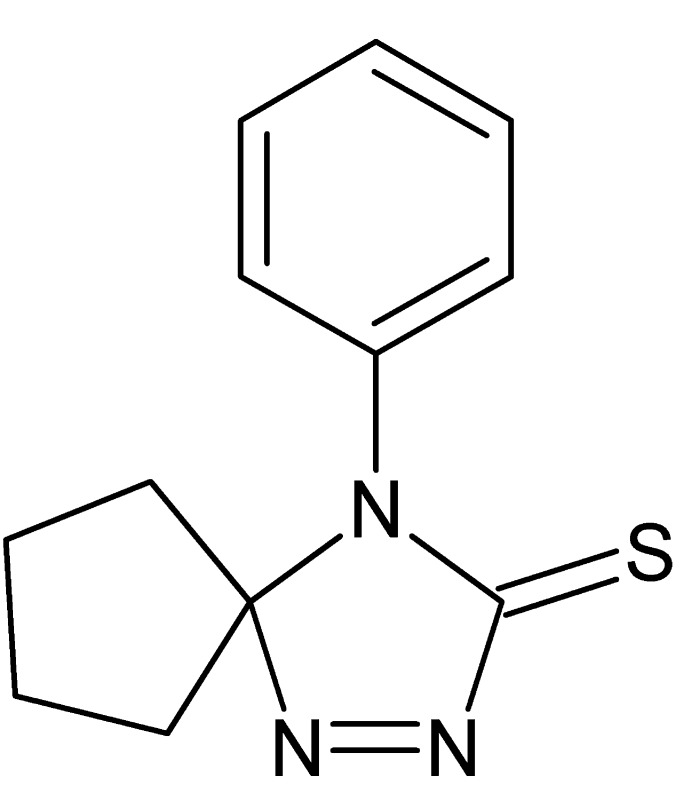


## Experimental   

### 

#### Crystal data  

C_12_H_13_N_3_S
*M* *_r_* = 231.32
Monoclinic, 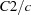

*a* = 11.4780 (12) Å
*b* = 12.0452 (12) Å
*c* = 17.0439 (17) Å
β = 101.3060 (14)°
*V* = 2310.7 (4) Å^3^
*Z* = 8
Mo *K*α radiation
μ = 0.26 mm^−1^
*T* = 150 K
0.15 × 0.13 × 0.12 mm

#### Data collection  

Bruker SMART APEX CCD diffractometer
Absorption correction: multi-scan (*TWINABS*; Sheldrick, 2009 ▶) *T*_min_ = 0.96, *T*_max_ = 0.97
29725 measured reflections
29725 independent reflections
21519 reflections with *I* > 2σ(*I*)
*R*_int_ = 0.050

#### Refinement  

*R*[*F* ^2^ > 2σ(*F* ^2^)] = 0.059
*wR*(*F* ^2^) = 0.168
*S* = 1.05
29725 reflections
146 parameters
44 restraints
H-atom parameters constrained
Δρ_max_ = 0.62 e Å^−3^
Δρ_min_ = −0.65 e Å^−3^

### 

Data collection: *APEX2* (Bruker, 2013 ▶); cell refinement: *SAINT* (Bruker, 2013 ▶); data reduction: *SAINT*; program(s) used to solve structure: *SHELXTL* (Sheldrick, 2008*b* ▶); program(s) used to refine structure: *SHELXL2014* (Sheldrick, 2008*b* ▶); molecular graphics: *DIAMOND* (Brandenburg & Putz, 2012 ▶) and *ORTEP-3 for Windows* (Farrugia, 2012 ▶); software used to prepare material for publication: *SHELXTL*.

## Supplementary Material

Crystal structure: contains datablock(s) global, I. DOI: 10.1107/S1600536814005418/rz5108sup1.cif

Structure factors: contains datablock(s) I. DOI: 10.1107/S1600536814005418/rz5108Isup2.hkl

Supporting information file. DOI: 10.1107/S1600536814005418/rz5108Isup3.cml

CCDC reference: 990878

Additional supporting information:  crystallographic information; 3D view; checkCIF report

## Figures and Tables

**Fig. 1.**
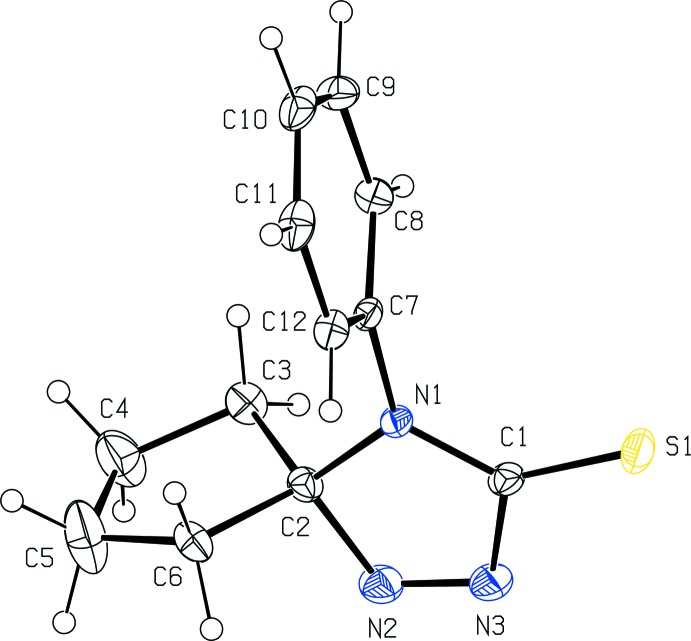
Perspective view of the title molecule with 30% displacement ellipsoids.

## supplementary crystallographic information

### 1. Comment

Heterocyclic spirocompounds are an important class of chemicals due to their great applications in medicinal and industrial fields. Beside the wide spectrum of their biological activities such as antibacterial agents (Sar *et al.*, 2006; Park *et al.*, 2007), anti-dermatitis agents (Nakao *et al.*, 2008), anticonvulsant agents (Obniska *et al.*, 2006; Kamiński *et al.*, 2008; Obniska *et al.*, 2006), anticancer agents (Chin *et al.*, 2008; Wang *et al.*, 2007), antimicrobial agents (Pawar *et al.*, 2009; Thadhaney *et al.*, 2010), anti-tuberculosis agents (Chande *et al.*, 2005), and recently as anti-oxidants (Shimakawa *et al.*, 2003; Sarma *et al.*, 2010), they act also as pesticides (Rongbao *et al.*, 2009), antifungal agents (Hu *et al.*, 2006), electroluminescent devices (Méhes *et al*., 2012) and laser dyes (Billah *et al.*, 2008). We were inspired by these findings to synthesize the title compound as part of our on-going study on synthesis and biological activity of spirocompound-based triazole derivatives.

The five-membered cyclopentane ring (C2–C6) of the title compound (I, Fig. 1), adopts a half-chair conformation with the puckering parameters (Cremer & Pople, 1975) of Q(2) = 0.250 (3) Å and φ(2) = 191.9 (9)°. The dihedral angle between the mean plane of the 4,5-dihydro-3*H*-1,2,4-triazole-3-thione ring (N1–N3/C1/C2) and the phenyl ring (C7–C12) is 85.49 (14)° with the S1 atom 0.046 (1) Å out of the former plane.

All bond lengths and bond angles in (I) are comparable with those for the similar compound *"4-Phenyl-1,2,4-triazaspiro[4.5]dec-1-ene-3-thione"* that we have reported previously (Akkurt *et al.*, 2013). The crystal structure is stabilized only by van der Waals interactions.

### 2. Experimental

The title compound was prepared according to our previous reported method (Akkurt *et al.* 2013). Orange block crystals suitable for X-ray diffraction were obtained from ethylacetate solution of I at room temperature (m.p. 455 – 457 K).

### 3. Refinement

The crystal used proved to be twinned by a 180° rotation about *c** (*CELL_NOW*, Sheldrick, 2008*a*) and the final structure was refined as a 2-component twin with a refined value of the minor twin fraction of 0.12203 (18). All H atoms were placed in geometrically idealized positions and constrained to ride on their parent atoms with O—H = 0.85 Å, N—H = 0.88 Å, C—H = 0.95 Å and 0.98 Å, with *U*_iso_(H) = 1.5 *U*_iso_(C) for methyl H atoms and *U*_iso_(H) = 1.2 *U*_iso_(C,*N*,*O*) for other H atoms. Restraints (DELU instructions in SHELXL-97) were used to reduce the components of the anisotropic displacement parameters of all atoms along the chemical bonds.

### Crystal data

**Table:**

C_12_H_13_N_3_S	*F*(000) = 976
*M**_r_* = 231.32	*D*_x_ = 1.330 Mg m^−^^3^
Monoclinic, *C*2/*c*	Mo *K*α radiation, λ = 0.71073 Å
Hall symbol: -C 2yc	Cell parameters from 8834 reflections
*a* = 11.4780 (12) Å	θ = 2.4–29.1°
*b* = 12.0452 (12) Å	µ = 0.26 mm^−^^1^
*c* = 17.0439 (17) Å	*T* = 150 K
β = 101.3060 (14)°	Block, orange
*V* = 2310.7 (4) Å^3^	0.15 × 0.13 × 0.12 mm
*Z* = 8	

### Data collection

**Table:**

Bruker SMART APEX CCD diffractometer	29725 independent reflections
Radiation source: fine-focus sealed tube	21519 reflections with *I* > 2σ(*I*)
Graphite monochromator	*R*_int_ = 0.050
Detector resolution: 8.3660 pixels mm^-1^	θ_max_ = 29.2°, θ_min_ = 2.4°
φ and ω scans	*h* = −15→15
Absorption correction: multi-scan (*TWINABS*; Sheldrick, 2009)	*k* = −16→16
*T*_min_ = 0.96, *T*_max_ = 0.97	*l* = −23→23
29725 measured reflections	

### Refinement

**Table:**

Refinement on *F*^2^	44 restraints
Least-squares matrix: full	Hydrogen site location: inferred from neighbouring sites
*R*[*F*^2^ > 2σ(*F*^2^)] = 0.059	H-atom parameters constrained
*wR*(*F*^2^) = 0.168	*w* = 1/[σ^2^(*F*_o_^2^) + (0.0674*P*)^2^ + 1.7625*P*] where *P* = (*F*_o_^2^ + 2*F*_c_^2^)/3
*S* = 1.05	(Δ/σ)_max_ < 0.001
29725 reflections	Δρ_max_ = 0.62 e Å^−^^3^
146 parameters	Δρ_min_ = −0.65 e Å^−^^3^

### Special details

**Table:**

Geometry. Bond distances, angles *etc*. have been calculated using the rounded fractional coordinates. All su's are estimated from the variances of the (full) variance-covariance matrix. The cell e.s.d.'s are taken into account in the estimation of distances, angles and torsion angles
Refinement. Refinement on *F*^2^ for ALL reflections except those flagged by the user for potential systematic errors. Weighted *R*-factors *wR* and all goodnesses of fit *S* are based on *F*^2^, conventional *R*-factors *R* are based on *F*, with *F* set to zero for negative *F*^2^. The observed criterion of *F*^2^ > σ(*F*^2^) is used only for calculating -*R*-factor-obs *etc*. and is not relevant to the choice of reflections for refinement. *R*-factors based on *F*^2^ are statistically about twice as large as those based on *F*, and *R*-factors based on ALL data will be even larger.

### Fractional atomic coordinates and isotropic or equivalent isotropic displacement parameters (Å^2^)

**Table:**

	*x*	*y*	*z*	*U*_iso_*/*U*_eq_	
S1	0.33502 (7)	0.94435 (6)	1.03564 (4)	0.0358 (2)	
N1	0.42067 (18)	0.80829 (17)	0.93431 (12)	0.0221 (6)	
N2	0.5566 (2)	0.7153 (2)	1.02559 (14)	0.0355 (8)	
N3	0.5050 (2)	0.7828 (2)	1.06315 (14)	0.0364 (8)	
C1	0.4168 (2)	0.8470 (2)	1.00689 (15)	0.0259 (8)	
C2	0.5122 (2)	0.7230 (2)	0.93878 (15)	0.0252 (7)	
C3	0.4680 (2)	0.6091 (2)	0.90472 (18)	0.0321 (9)	
C4	0.5715 (3)	0.5557 (3)	0.8784 (3)	0.0627 (14)	
C5	0.6575 (4)	0.6450 (3)	0.8686 (3)	0.0695 (16)	
C6	0.6150 (2)	0.7526 (2)	0.89633 (17)	0.0321 (9)	
C7	0.3486 (2)	0.8458 (2)	0.86065 (14)	0.0214 (7)	
C8	0.2459 (2)	0.7876 (2)	0.82843 (16)	0.0289 (8)	
C9	0.1807 (3)	0.8195 (3)	0.75431 (17)	0.0374 (9)	
C10	0.2175 (3)	0.9075 (3)	0.71427 (16)	0.0389 (10)	
C11	0.3174 (3)	0.9671 (2)	0.74808 (16)	0.0358 (9)	
C12	0.3844 (2)	0.9370 (2)	0.82186 (16)	0.0280 (8)	
H3A	0.44130	0.56350	0.94620	0.0380*	
H3B	0.40080	0.61790	0.85890	0.0380*	
H4A	0.60970	0.50130	0.91890	0.0750*	
H4B	0.54480	0.51630	0.82710	0.0750*	
H5A	0.66400	0.65090	0.81160	0.0840*	
H5B	0.73710	0.62700	0.90030	0.0840*	
H6A	0.58690	0.80240	0.85030	0.0380*	
H6B	0.67980	0.79020	0.93380	0.0380*	
H8	0.22050	0.72710	0.85660	0.0350*	
H9	0.11060	0.78020	0.73130	0.0450*	
H10	0.17400	0.92750	0.66290	0.0470*	
H11	0.34040	1.02950	0.72060	0.0430*	
H12	0.45330	0.97790	0.84520	0.0340*	

### Atomic displacement parameters (Å^2^)

**Table:**

	*U*^11^	*U*^22^	*U*^33^	*U*^12^	*U*^13^	*U*^23^
S1	0.0424 (4)	0.0413 (4)	0.0254 (4)	0.0049 (3)	0.0109 (3)	−0.0062 (3)
N1	0.0225 (10)	0.0259 (11)	0.0175 (10)	0.0026 (9)	0.0027 (8)	0.0011 (8)
N2	0.0318 (13)	0.0438 (14)	0.0288 (13)	0.0038 (11)	0.0009 (10)	0.0069 (11)
N3	0.0341 (13)	0.0498 (15)	0.0227 (12)	0.0028 (12)	−0.0006 (10)	0.0044 (11)
C1	0.0259 (13)	0.0331 (14)	0.0181 (12)	−0.0035 (11)	0.0030 (10)	0.0014 (10)
C2	0.0227 (12)	0.0273 (13)	0.0252 (13)	0.0043 (10)	0.0040 (11)	0.0036 (10)
C3	0.0322 (15)	0.0253 (14)	0.0392 (16)	0.0011 (11)	0.0082 (13)	0.0029 (12)
C4	0.042 (2)	0.050 (2)	0.101 (3)	−0.0047 (16)	0.026 (2)	−0.026 (2)
C5	0.070 (3)	0.049 (2)	0.107 (3)	−0.008 (2)	0.060 (3)	−0.016 (2)
C6	0.0243 (13)	0.0354 (16)	0.0385 (16)	0.0022 (11)	0.0110 (12)	0.0045 (12)
C7	0.0243 (12)	0.0245 (13)	0.0151 (11)	0.0056 (10)	0.0030 (9)	−0.0012 (9)
C8	0.0264 (13)	0.0304 (14)	0.0291 (14)	0.0015 (11)	0.0038 (11)	0.0004 (11)
C9	0.0290 (15)	0.0458 (18)	0.0329 (16)	0.0075 (13)	−0.0051 (12)	−0.0081 (13)
C10	0.0472 (18)	0.0466 (18)	0.0201 (13)	0.0245 (15)	−0.0004 (13)	−0.0006 (12)
C11	0.0524 (18)	0.0318 (16)	0.0253 (14)	0.0142 (14)	0.0130 (13)	0.0076 (11)
C12	0.0329 (14)	0.0266 (14)	0.0252 (14)	0.0027 (11)	0.0075 (11)	−0.0001 (10)

### Geometric parameters (Å, º)

**Table:**

S1—C1	1.636 (3)	C10—C11	1.380 (5)
N1—C1	1.331 (3)	C11—C12	1.387 (4)
N1—C2	1.461 (3)	C3—H3A	0.9900
N1—C7	1.434 (3)	C3—H3B	0.9900
N2—N3	1.253 (3)	C4—H4A	0.9900
N2—C2	1.470 (3)	C4—H4B	0.9900
N3—C1	1.470 (3)	C5—H5A	0.9900
C2—C3	1.537 (3)	C5—H5B	0.9900
C2—C6	1.542 (3)	C6—H6A	0.9900
C3—C4	1.495 (4)	C6—H6B	0.9900
C4—C5	1.491 (6)	C8—H8	0.9500
C5—C6	1.494 (5)	C9—H9	0.9500
C7—C8	1.389 (3)	C10—H10	0.9500
C7—C12	1.385 (3)	C11—H11	0.9500
C8—C9	1.390 (4)	C12—H12	0.9500
C9—C10	1.371 (5)		
			
C1—N1—C2	110.7 (2)	C4—C3—H3A	111.00
C1—N1—C7	125.8 (2)	C4—C3—H3B	111.00
C2—N1—C7	123.6 (2)	H3A—C3—H3B	109.00
N3—N2—C2	111.6 (2)	C3—C4—H4A	110.00
N2—N3—C1	110.0 (2)	C3—C4—H4B	110.00
S1—C1—N1	130.9 (2)	C5—C4—H4A	110.00
S1—C1—N3	122.94 (19)	C5—C4—H4B	110.00
N1—C1—N3	106.1 (2)	H4A—C4—H4B	108.00
N1—C2—N2	101.58 (19)	C4—C5—H5A	110.00
N1—C2—C3	115.3 (2)	C4—C5—H5B	110.00
N1—C2—C6	114.9 (2)	C6—C5—H5A	110.00
N2—C2—C3	110.3 (2)	C6—C5—H5B	110.00
N2—C2—C6	109.9 (2)	H5A—C5—H5B	108.00
C3—C2—C6	104.8 (2)	C2—C6—H6A	111.00
C2—C3—C4	105.9 (2)	C2—C6—H6B	111.00
C3—C4—C5	107.8 (3)	C5—C6—H6A	111.00
C4—C5—C6	109.0 (3)	C5—C6—H6B	111.00
C2—C6—C5	106.0 (2)	H6A—C6—H6B	109.00
N1—C7—C8	119.1 (2)	C7—C8—H8	121.00
N1—C7—C12	119.6 (2)	C9—C8—H8	121.00
C8—C7—C12	121.3 (2)	C8—C9—H9	120.00
C7—C8—C9	118.9 (2)	C10—C9—H9	120.00
C8—C9—C10	120.2 (3)	C9—C10—H10	120.00
C9—C10—C11	120.4 (3)	C11—C10—H10	120.00
C10—C11—C12	120.7 (2)	C10—C11—H11	120.00
C7—C12—C11	118.4 (2)	C12—C11—H11	120.00
C2—C3—H3A	111.00	C7—C12—H12	121.00
C2—C3—H3B	111.00	C11—C12—H12	121.00
			
C2—N1—C1—S1	177.9 (2)	N2—N3—C1—N1	1.5 (3)
C2—N1—C1—N3	−2.4 (3)	N1—C2—C3—C4	153.1 (3)
C7—N1—C1—S1	−0.4 (4)	N2—C2—C3—C4	−92.6 (3)
C7—N1—C1—N3	179.3 (2)	C6—C2—C3—C4	25.7 (3)
C1—N1—C2—N2	2.4 (3)	N1—C2—C6—C5	−149.9 (3)
C1—N1—C2—C3	121.7 (2)	N2—C2—C6—C5	96.3 (3)
C1—N1—C2—C6	−116.2 (2)	C3—C2—C6—C5	−22.2 (3)
C7—N1—C2—N2	−179.3 (2)	C2—C3—C4—C5	−19.6 (4)
C7—N1—C2—C3	−60.0 (3)	C3—C4—C5—C6	5.6 (5)
C7—N1—C2—C6	62.1 (3)	C4—C5—C6—C2	10.6 (4)
C1—N1—C7—C8	−96.5 (3)	N1—C7—C8—C9	−175.6 (2)
C1—N1—C7—C12	85.5 (3)	C12—C7—C8—C9	2.5 (4)
C2—N1—C7—C8	85.5 (3)	N1—C7—C12—C11	175.7 (2)
C2—N1—C7—C12	−92.6 (3)	C8—C7—C12—C11	−2.3 (4)
C2—N2—N3—C1	0.1 (3)	C7—C8—C9—C10	−0.4 (4)
N3—N2—C2—N1	−1.5 (3)	C8—C9—C10—C11	−1.9 (5)
N3—N2—C2—C3	−124.2 (2)	C9—C10—C11—C12	2.1 (5)
N3—N2—C2—C6	120.7 (2)	C10—C11—C12—C7	0.0 (4)
N2—N3—C1—S1	−178.8 (2)		
